# Incidence and predictors of post-thrombotic syndrome in patients with proximal DVT in a real-world setting: findings from the GARFIELD-VTE registry

**DOI:** 10.1007/s11239-023-02895-7

**Published:** 2023-11-06

**Authors:** Paolo Prandoni, Sylvia Haas, Meg E. Fluharty, Sebastian Schellong, Harry Gibbs, Eric Tse, Marc Carrier, Barry Jacobson, Hugo ten Cate, Elizaveta Panchenko, Peter Verhamme, Karen Pieper, Gloria Kayani, Lord A. Kakkar, the GARFIELD-VTE investigators, Nik Abdullah, Akihiko Abiko, Juan Abril, David Acevedo, Taylan Adademir, David Adler, Walter Ageno, Giancarlo Agnelli, Mostafa Ahmed, Ahmet Aksoy, Serir Aktogu, Gholam Ali, Raz Alikhan, Gregory Allen, Pantep Angchaisuksiri, Sevestre Antoinette, Amy Arouni, Addala Azeddine, Tarek Azim, Wilfried Backer, Yohan Balthazar, Soo Bang, Martin Banyai, Olga Barbarash, Marcelo Barrionuevo, Mostafa Bary, Bektas Battaloglu, W. Bax, Terriat Béatrice, Steffen Behrens, Dmitry Belenky, Juan Benitez, Mario Berli, Peuch Bernadette, Andrea Berni, Michiel Betsbrugge, Adriaan Beyers, Abraham Bezuidenhout, Claude Bidi, Peter Bilderling, Laure Binet, Tina Biss, Luis Blasco, Erwin Blessing, Peter Blombery, Julio Bono, Karin Boomars, Juree Boondumrongsagoon, Lohana Borges, Manuel Bosch, Louis Botha, Henri Bounameaux, Tim Boussy, Margaret Bowers, Mikhail Boyarkin, Cornelia Brauer, Kate Burbury, Hana Burianova, Yuriy Burov, Cas Cader, Reto Canevascini, Luc Capiau, Roberto Cappelli, Boulon Carine, Marc Carrier, Abu Carrim, Patrick Carroll, Tomas Casabella, Hugo Cate, Marco Cattaneo, Vladimir Cech, Luis Cervera, Seung Cha, Joseph Chacko, Kuan Chang, Kanchana Chansung, Ting Chao, Anoop Chauhan, Sunee Chayangsu, Mariam Chetanachan, Lee Chew, Chern Chiang, Kuan Chiu, Won Choi, Ponchaux Christian, Brousse Christophe, Seinturier Christophe, Sanjeev Chunilal, Amanda Clark, Abdurrahim Colak, João Correa, Benilde Cosmi, Franco Cosmi, Zdenek Coufal, Desmond Creagh, Leone Cristina, Carlos Cuneo, Garcia Dalmau, Garrigues Damien, Armando D’Angelo, Harald Darius, Sudip Datta, Adriaan Dees, Mohamed Dessoki, Carlos Diaz, Enrique Diaz, Emre Dogan, Brisot Dominique, Elkouri Dominique, Stephan Dominique, Servaas Donders, Dmitry Dorokhov, Johan Duchateau, Norberto Duda, Grace Eddie, Hallah Elali, Hesham ElDin, Chevrier Elisa, Messas Emmanuel, Barbara Erdelyi, Frans Erdkamp, Ehab Esheiba, Guillermo Esperón, Sherif Essameldin, Tamara Everington, Markus Faghih, Anna Falanga, Jose Fedele, Richard Ferkl, Alberto Fernandez, Manuel Fernandez, Piera Ferrini, Fabian Ferroni, Jose Filho, Mark Fixley, John Fletcher, Oscar Flores, Couturaud Francis, Bergmann Francois, Hendrik Franow, Amr Gad, Mohamed Gaffar, Mary Gaffney, Gregoire Gal, Javier Galvar, Angel Galvez, Marco Gamba, Gin Gan, Victor Gerdes, Hagen Gerofke, Harry Gibbs, Harinder Gogia, Ivan Gordeev, Shinya Goto, Sam Griffin, Christina Gris, Ernst Grochenig, Jaspal Gujral, Ozcan Gur, Orcun Gurbuz, Michel Gustin, Luis Guzman, Chung Ha, Ghassan Haddad, Dirk Hagemann, Philippe Hainaut, Muhammad Hameed, Terence Hart, Hatice Hasanoglu, Erman Hashas, Wilhelm Haverkamp, Desmurs Helene, Fitjerald Henry, Artur Herdy, Rika Herreweghe, Masao Hirano, Prahlad Ho, Wai Ho, Geert Hollanders, Miroslav Homza, Thomas Horacek, Chien Hsia, Chien Huang, Chi Huang, Chun Huang, Julian Humphrey, Beverley Hunt, Azlan Husin, Hun Hwang, Piriyaporn Iamsai, Manuel Ibarra, Davide Imberti, Mahe Isabelle, Selim Isbir, Barry Jacobson, Petr Jansky, Weihong Jiang, David Jimenez, Zhicheng Jing, Zhicheng Jing, Jin Joh, Gadel Kamalov, Junji Kanda, Masashi Kanemoto, Nonglak Kanitsap, Muhip Kanko, Kemal Karaarslan, Jeannine Kassis, Atsushi Kato, Andrey Kazakov, David Keeling, Reinhold Keim, Allan Kelly, Mohamed Khan, Bonnie Kho, Alexey Khotuntsov, Ho Kim, Igor Kim, JangYong Kim, Jin Kim, Moo Kim, Yang Kim, Ilker Kiris, Robert Klamroth, Andres Kleiban, Garry Klein, Katsuhiro Kondo, Martin Koretzky, Wolfgang Korte, Modise Koto, Firas Koura, Michael Kovacs, Vladimir Krasavin, Alan Krichell, Knut Kroeger, Ralf Kroening, Jiri Krupicka, Emre Kubat, Dusan Kucera, Shintaro Kuki, Jen Kuo, Jan Kvasnicka, Chi Kwok, JiHyun Kwon, Wen Lai, Pavel Lang, Jose Lara, Jiri Lastuvka, Holger Lawall, Michael Leahy, Jae Lee, Moon Lee, Raul Leon, Siwe Léopold, Michael Levy, Igor Libov, Wei Lin, Ann Lockman, Corrado Lodigiani, Irene Looi, Luciano López, Ab Loualidi, Charles Lunn, Canhua Luo, Thifhelimbilu Luvhengo, Shaun Maasdorp, Peter MacCallum, Andrew Machowski, Mujibur Majumder, Nisa Makruasi, Wagih Malek, Kubina Manuel, Pablo Marchena, Javier Marino, Rafael Martinez, Shunzo Matsuoka, Antonino Mazzone, Simon McRae, Stuart Mellor, Robert Mendes, Geno Merli, Antoni Mestre, Escande Michèle, Saskia Middeldorp, Raimundo Miranda, Ahmed Mohamed, Monniaty Mohamed, Marco Moia, Dorthe Møller, Serge Motte, Moustafa Moustafa, Nicola Mumoli, Yeung Mun, Michael Munch, Juan Muntaner, Bisher Mustafa, Pramook Mutirangura, Martin Myriam, Sang Na, Mohamed Nagib, Hiroaki Nakamura, Mashio Nakamura, Satoshi Nakazawa, Seung Nam, Bhavesh Natha, Falvo Nicolas, Jørn Nielsen, Lalita Norasetthada, Nordiana Nordin, Tontanai Numbenjapon, Ole Nyvad, Hans Ohler, Yasushi Ohnuma, Michael Olsen, Tomoya Onodera, Christian Opitz, Alisha Oropallo, Remedios Otero, Oztekin Oto, Jorge Paez, Elizaveta Panchenko, Félix Paredes, Jin Park, Yong Park, Nishen Paruk, Siriwimon Patanasing, Guillot Paul, Michel Pauw, Jose Peromingo, Dmitry Petrov, Walter Pharr, Georg Plassmann, George Platt, Ivo Podpera, Germain Poirier, Daniela Poli, Ettore Porreca, Domenico Prisco, Robert Prosecky, Jiri Pumprla, Herbert Raedt, Rapule Ratsela, Selma Raymundo, Raquel Reyes, Tim Reynolds, Luigi Ria, Ponlapat Rojnuckarin, Dirk Roux, Ayman Salem, Rita Santoro, Jose Saraiva, Jameela Sathar, Ismail Savas, Sebastian Schellong, Lilia Schiavi, Andor Schmidt, Renate Schmidt, Herman Schroe, Marlin Schul, Carsten Schwencke, David Scott, Gaurand Shah, Yoshisato Shibata, Jhih Shih, Hyeok Shim, Sherif Sholkamy, Kou Shyu, Rupesh Singh, Suaran Singh, Dirk Skowasch, Alison Slocombe, Clifford Smith, German Sokurenko, Mosaad Soliman, Susan Solymoss, Ik Song, Igor Sonkin, Joan Souto, Rudolf Spacek, Ilya Staroverov, Daniel Staub, Harry Striekwold, Markus Stuecker, Yuriy Subbotin, Igor Suchkov, Shenghua Sun, Jose Surinach, Tawatchai Suwanban, Koscál Svatopluk, Jaromira Svobodova, Mersel Tahar, Kensuke Takeuchi, Yasuhiro Tanabe, Isabel Tenorio, Sophie Testa, Daniel Theodoro, Hongyan Tian, Lidwine Tick, Luc Timmermans, Seng Ting, Eros Tiraferri, Cheng Toh, See Toh, Vladimir Tolstikhin, Jorge Toro, Jorge Toro, Alberto Tosetto, Berremeli Toufek, Bruno Trimarco, Eric Tse, Wei Tseng, Hatice Turker, Kwo Ueng, Esther Usandizaga, Kristel Vandenbosch, Jan Vanwelden, Peter Verhamme, Jiri Vesely, Beatrice Vesti, Pongtep Viboonjuntra, Oscar Vilamajo, Philippe Vleeschauwer, Haofu Wang, Shenming Wang, Chris Ward, Akinori Watanabe, Simon Watt, James Welker, Rachel Wells, Kwan Wern, Jan Westendorf, Richard White, Benedicte Wilson, Lily Wong, Raymond Wong, Somchai Wongkhantee, Chau Wu, Chih Wu, Cynthia Wu, Jinghua Yang, Zhenwen Yang, Zhongqi Yang, Celal Yavuz, Erik Yeo, Ho Yhim, Kai Yiu, Shuichi Yoshida, Winston Yoshida, Cesar Zaidman, Dmitry Zateyshchikov, Thomas Zeller, Stanislav Zemek, Lei Zhang, Weihua Zhang, Hong Zhu, Hesham Zidan, Brian Zidel, Konstantin Zrazhevskiy, Nadezhda Zubareva

**Affiliations:** 1 Arianna Foundation on Anticoagulation, Bologna, Italy; 2 Technical University of Munich, Munich, Germany; 3 Thrombosis Research Institute, London, UK; 4 Medical Department 2, Municipal Hospital Dresden, Dresden, Germany; 5 Department of General Medicine, Alfred Hospital, Melbourne, VIC Australia; 6 Department of Medicine, The University of Hong Kong, Queen Mary Hospital, Pok Fu Lam, Hong Kong; 7 Department of Medicine, Ottawa Hospital Research Institute at the University of Ottawa, Ottawa, ON Canada; 8 Department of Haematology and Molecular Medicine, University of the Witwatersrand, Johannesburg, South Africa; 9 Division of Vascular Medicine and Thrombosis Expertise Center, Department of Internal Medicine, Maastricht University Medical Center (MUMC+), Maastricht, The Netherlands; 10 National Medical Research Center of Cardiology Named After Academician E.I. Chazov, Moscow, Russia; 11 Department of Cardiovascular Sciences, University of Leuven, Leuven, Belgium

**Keywords:** Post-thrombotic syndrome, Venous thromboembolism, Deep vein thrombosis, GARFIELD-VTE, Registry

## Abstract

Although substantial progress has been made in the pathophysiology and management of the post-thrombotic syndrome (PTS), several aspects still need clarification. Among them, the incidence and severity of PTS in the real world, the risk factors for its development, the value of patient’s self-evaluation, and the ability to identify patients at risk for severe PTS. Eligible participants (n = 1107) with proximal deep-vein thrombosis (DVT) from the global GARFIELD-VTE registry underwent conventional physician’s evaluation for PTS 36 months after diagnosis of their DVT using the Villalta score. In addition, 856 patients completed a Villalta questionnaire at 24 months. Variable selection was performed using stepwise algorithm, and predictors of severe PTS were incorporated into a multivariable risk model. The optimistic adjusted c-index was calculated using bootstrapping techniques. Over 36-months, 27.8% of patients developed incident PTS (mild in 18.7%, moderate in 5.7%, severe in 3.4%). Patients with incident PTS were older, had a lower prevalence of transient risk factors of DVT and a higher prevalence of persistent risk factors of DVT. Self-assessment of overall PTS at 24 months showed an agreement of 63.4% with respect to physician’s evaluations at 36 months. The severe PTS multivariable model provided an optimistic adjusted c-index of 0.68 (95% CI 0.59–0.77). Approximately a quarter of DVT patients experienced PTS over 36 months after VTE diagnosis. Patient’s self-assessment after 24 months provided added value for estimating incident PTS over 36 months. Multivariable risk analysis allowed good discrimination for severe PTS.

**Supplementary Information:**

The online version contains supplementary material available at 10.1007/s11239-023-02895-7.

## Highlights

- Incidence and severity of post-thrombotic syndrome (PTS) is lower than previously reported.
- There is fair agreement between PTS self-assessment at 24 months and physician’s evaluation at 36 months.
- A multivariable risk analysis allowed good discrimination for severe PTS.

## Introduction

Post-thrombotic syndrome (PTS) is by far the most frequent long-term complication of deep-vein thrombosis (DVT) of the lower extremities. Based on the results of prospective cohort studies and randomized clinical trials, it is expected to occur in 40 to 50% of patients with a first episode of proximal DVT, and in 8–10% is serious enough to severely impair patient’s quality of life [1–4]. It is generally defined and classified according to the Villalta score [5–7].

Although substantial progress has been made in the pathophysiology and management of this long-term complication of DVT, several aspects still need clarification [8]. Among them, the incidence and severity of PTS in the real world, the risk factors for its development, the ability to predict the development of severe PTS, and the value of patient’s self-evaluation, particularly desirable in circumstances like the current pandemic.

Global Anticoagulant Registry in the FIELD-Venous Thromboembolism (GARFIELD-VTE) is a prospective, non-interventional, global registry of over 10,000 patients with VTE recruited worldwide [9]. As in an unselected subgroup of patients referring with DVT, careful information on the long-term development of PTS was collected, we had the opportunity to address some clinically relevant questions that are still unanswered.

The main purposes of our investigation were: to determine the 36-month incidence of overall and severe PTS in a broad series of unselected patients belonging to the GARFIELD-VTE registry; to assess the risk factors of PTS development; to assess the value of patient’s self-evaluation via the Villalta score after 24 months; and to test the discriminatory ability of a multivariable model for the risk of severe PTS. To ensure these findings were comparable with previously published data, only patients with proximal DVT were included in the study analysis.

## Materials and methods

GARFIELD-VTE included more than 10,000 registered participants with recent diagnosis of VTE over a 3-year period and collected prospective data on clinical outcomes, treatment patterns and risk factors. The registry was funded by an unrestricted research grant from Bayer AG, and the ongoing work is supported by the Thrombosis Research Institute. At the end of the follow-up period, in patients recruited for lower limb DVT, alone or associated with pulmonary embolism (PE), physicians who had agreed a priori on this program were asked to make a PTS assessment based on the Villalta score. The presence of five leg symptoms (i.e. pain, cramps, heaviness, paresthesia and pruritus) and six objective signs (i.e. pretibial edema, induration of the skin, hyperpigmentation, redness, venous ectasia, and pain during calf compression) was scored. For each item, a score of 0 (not present) up to 3 (most severe) was assigned, using the contralateral unaffected leg as reference. The presence of a score of 0 to 4 indicated no PTS, 5 to 9, mild PTS, and 10 to 14, moderate PTS, while a score of 15 or more or the presence of skin ulcer indicated severe PTS [5].

One year earlier, a patient’s self-assessment was scheduled in consenting subjects with the help of a questionnaire (sent by mail) enquiring the presence and severity of the subjective symptoms and objective findings captured by the Villalta score (all but skin ulcer). For this purpose, the 11 parameters of the Villalta Score were translated into lay language and given to patients for self-examination and answering the questions (Table 1). For each complaint, patients were requested to quantify the burden using a Likert scale from 0 (not present) to 10 (most severe). Each question in the questionnaire was converted to the original score using the following rule: 0 = absent; 1 to 3 = 1 point; 4 to 7 = 2 points; 8 to 11 = 3 points. An additional question enquired the pre-existence of complaints before the DVT episode. If this was the case, the related sign or symptom was only computed if according to the patient’s opinion it had significantly worsened. To be consistent with the Villalta score, the presence of an overall score of 5 to 9 indicated mild PTS, of 10 to 14 moderate PTS, while a score of 15 or more indicated severe PTS. The original Villalta score can be found in the supplementary material (Supplementary Table 1).

**Table 1:**
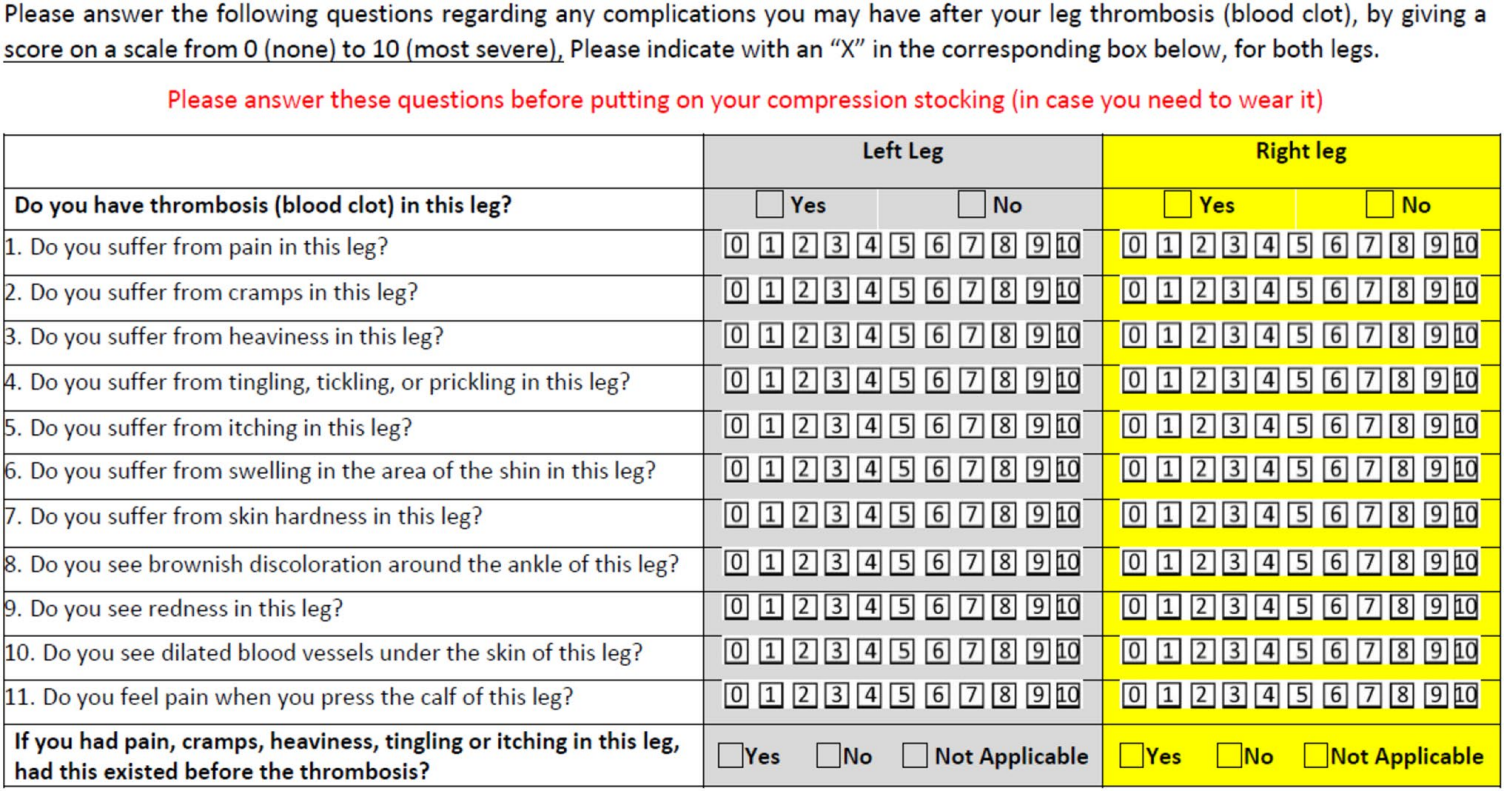
The questionnaire composed of original Villalta score parameters and translated into lay language

The original Villalta score for each question is 0–3. The 0–10 questions below are mapped to the 0–3 Villalta scores as 0 = 0 (none) 1 to 3 = 1 (mild) 4 to 7 = 2 (moderate) and 8 to 10 = 3 (severe)

Contingency tables comparing the frequency distribution of the derived PTS diagnosis (none, mild, moderate, severe) were created. The agreement between the patient’s self-assessment and the physician assessment was quantified by the proportion of identical PTS diagnosis from the total.

To test the discriminatory ability of a multivariable model for the risk of severe PTS, a multivariable logistic regression model was calculated according to standard methods. The patient variables at baseline to be included in the model were the following: (1) patient demographics: age, gender, ethnicity, BMI and smoking, (2) treatment modalities (conventional and direct oral anticoagulants); (3) the prescription of elastic compression stockings; (4) provoking factors of VTE (within 3 months before diagnosis): acute medical illness, hospitalization, long-haul travelling, trauma of the lower limb, surgery, and active cancer; (5) special risk factors associated with VTE: active cancer; (6) predisposing risk factors: chronic heart failure, chronic immobilization, family history of VTE, history of cancer, known thrombophilia, prior episode of DVT, and renal insufficiency.

The Area Under the Receiver Operating Characteristic (ROC) curve (AUC) was calculated as a measure of the ability of the model to distinguish between patients who developed PTS from those that did not. A calibration plot displaying the predicted event rate against the observed rates was constructed. The predicted event rates were divided into quintiles and the mean event rate in each quintile was calculated and plotted against the mean observed event rate in each decile. Finally, the discrimination performance of the final model was evaluated through c-index (AUC) by adjusting for optimism using bootstrapping. The analysis was conducted using the statistical programme SAS.

## Results

### Patients and development of PTS

**Fig. 1:**
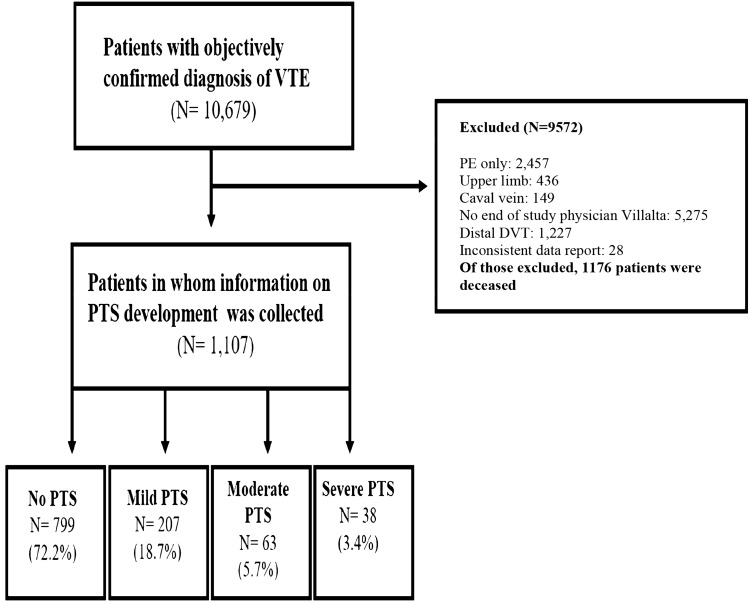
Flow diagram showing patient population and incidence of 36-month overall and severe PTS. *PTS* post-thrombotic syndrome, *PE* pulmonary embolism, *VTE* venous thromboembolism

Of the 10 679 patients with acute VTE that were recruited in the GARFIELD-VTE registry between May 2014 and January 2017, 2457 were excluded because of clinical presentation with isolated PE. A further 436 were excluded due to upper limb DVT (N = 436), caval vein thrombosis (N = 149), isolated distal DVT (N = 1227), died during the study enrolment (N = 1175), or lacked of end-of-study physician assessment (N = 4082). A final 28 participants were excluded due to inconsistent data provided. Accordingly, 1107 patients were recruited in the current investigation. Based on the Villalta score, PTS was detected in 308 patients (27.8%), and was mild in 207 (18.7%), moderate in 63 (5.7%), and severe in the remaining 38 (3.4%) (Fig. 1).

### Risk factors of PTS

The main demographic and clinical characteristics of the recruited patients including their persistent and provoking risk factors are shown in Table 2. Patients with PTS were on average older and were more likely to be Caucasian. Patients with PTS also had a lower prevalence of long-haul travelling among the provoking risk factors; and were more likely to have chronic heart failure and chronic immobilization among the predisposing risk factors.

**Table 2:** Demographic and clinical characteristics of the overall population according to PTS status

Variable	Statistics	Level	Post thrombotic syndrome (PTS)		p value*
			Yes N = 308	No N = 799	
Demographics					
Age (years)	N		308	799	**0.009**
	Mean (SD)		58.2 (16.7)	55.3 (16.3)	
	Median (Q1; Q3)		59.6 (45.8;69.7)	56.6 (42.7;67.6)	
BMI (kg/m^2^)	N		294	761	0.153
	Mean (SD)		29.4 (6.1)	28.8 (6.3)	
	Median (Q1; Q3)		28.7 (25.4;32.4)	27.8 (24.7;31.9)	
Gender		Male	160 (51.9)	437 (54.7)	0.412
		Female	148 (48.1)	362 (45.3)	
Ethnicity/race		Asian	14 (5.1)	75 (10.0)	** < 0.001**
		Black	9 (3.3)	27 (3.6)	
		Caucasian	234 (86.0)	543 (72.1)	
		Multi-racial	3 (1.1)	3 (0.4)	
		Other	12 (4.4)	105 (13.9)	
Smoking status		Never smoker	191 (63.2)	506 (64.1)	0.725
		Ex-smoker	53 (17.5)	147 (18.6)	
		Current smoker	58 (19.2)	136 (17.2)	
Treatments					
		Parenteral therapy only	34 (11.0)	101 (12.7)	0.073
		Parenteral therapy + VKA	94 (30.5)	263 (33.0)	
		VKA only	12 (3.9)	39 (4.9)	
		DOAC only	100 (32.5)	285 (35.7)	
		Parenteral therapy + DOAC	62 (20.1)	101 (12.7)	
		Other AC	2 (0.6)	3 (0.4)	
		No AC treatment	4 (1.3)	6 (0.8)	
		Compression therapy	200 (64.9)	517 (64.7)	0.943
Provoking risk factors					
		Acute medical illness	19 (6.2)	42 (5.3)	0.551
		Hospitalisation	27 (8.8)	65 (8.1)	0.733
		Long-haul travelling	7 (2.3)	43 (5.4)	**0.026**
		Trauma of the lower limb	24 (7.8)	89 (11.1)	0.099
		Surgery	27 (8.8)	68 (8.5)	0.892
		Active cancer	6 (1.9)	29 (3.6)	0.152
Persistent/predisposing risk factors					
		Chronic heart failure	15 (4.9)	11 (1.4)	** < 0.001**
		Chronic immobilisation	25 (8.1)	39 (4.9)	**0.039**
		Family history of VTE	22 (7.1)	40 (5.0)	0.166
		History of cancer	25 (8.1)	60 (7.5)	0.734
		Known thrombophilia	13 (4.2)	28 (3.5)	0.572
		Prior episode of DVT and/or PE	83 (26.9)	135 (16.9)	** < .001**
		Renal insufficiency	6 (1.9)	22 (2.8)	0.444

Numbers in parenthesis indicate percentages, unless otherwise specified

*PTS* post-thrombotic syndrome, *BMI* body mass index, *DVT* deep-vein thrombosis, *PE* pulmonary embolism, *VTE* venous thromboembolism

*The parametric p value is calculated by ANOVA for numerical covariates and chi-square test for categorical covariates and is indicated in bold

### Patient’s self-assessment

**Fig. 2:**
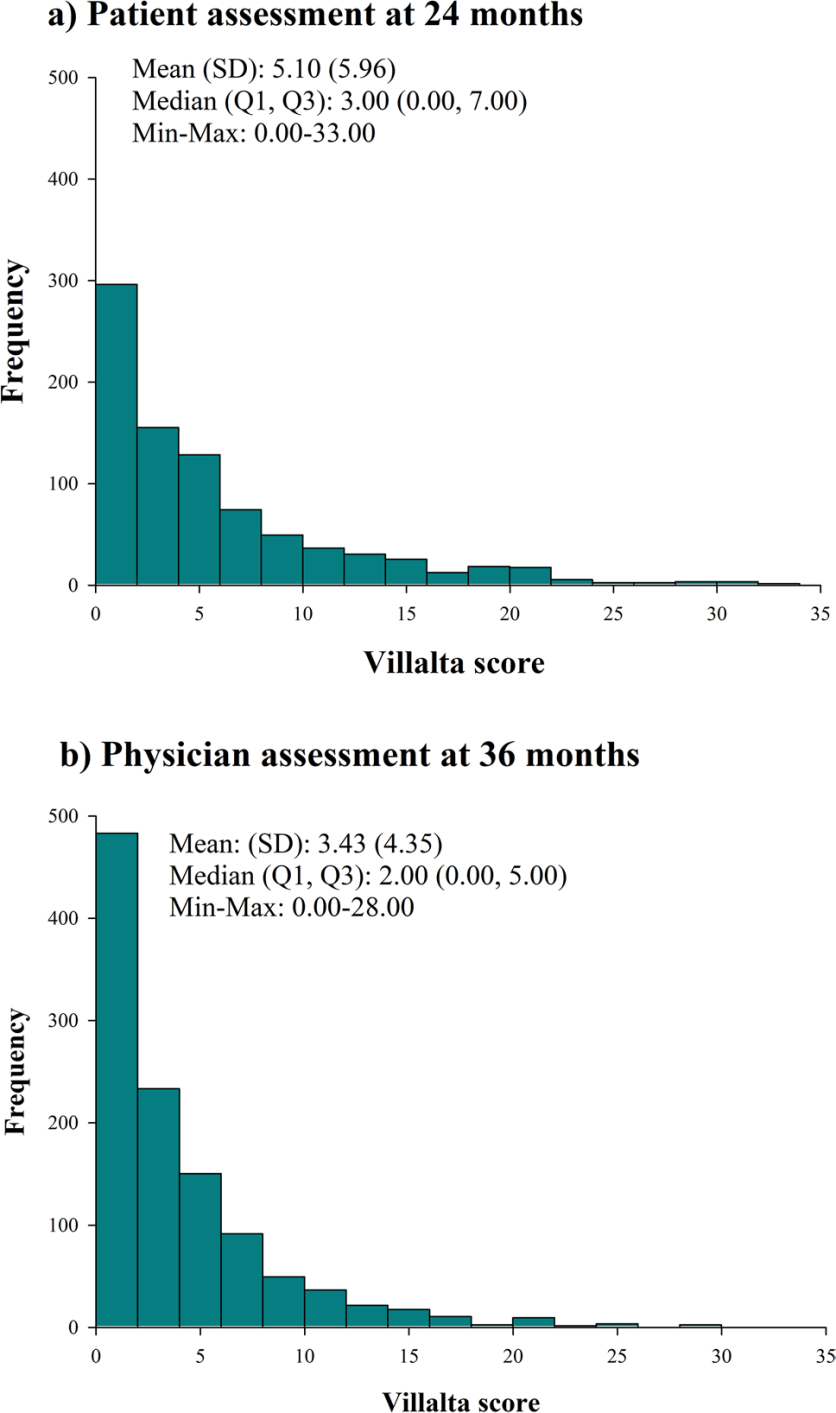
Histogram of Villalta scores from **a** patient assessment at 24 months* and **b** physician assessment at 36 months. **a** *SD* standard deviation; *Q* quartile. *In some cases, patient assessment took place at 6 months when a 24-month assessment was not feasible. **b** *SD* standard deviation, *Q* quartile

The self-assessment of overall PTS at 24 months was available in 856 of the 1107 patients (77%) patients. As shown in Table 3 with respect to physician’s evaluation at 36 months, patient’s self-assessment at 24 months had an agreement of 63.4% with a weighted kappa of 0.37 (0.31,0.42). The median (Q1, Q2) and Mean (SD) Villalta score for patients’ self-assessment at 24 months and physician’s assessment at 36 months have been shown in Fig. 2.

**Table 3:** Contingency table comparing patient (24 month) and physician (36 month) Villalta scores

			Physician Villalta 36 months				
			None	Mild	Moderate	Severe	Total
*Frequency*	Patient Villalta 24 months	None	445	72	10	3	530
*Percent*			52	8.41	1.17	0.35	61.9
*Row percent*			84	13.6	1.89	0.57	
*Column percent*			74.2	40.9	19.23	10.71	
		Mild	100	60	9	3	172
			11.7	7.01	1.05	0.35	20.1
			58.1	34.9	5.23	1.74	
			16.7	34.1	17.31	10.71	
		Moderate	28	26	17	7	78
			3.27	3.04	1.99	0.82	9.11
			35.9	33.3	21.79	8.97	
			4.67	14.8	32.69	25	
		Severe	27	18	16	15	76
			3.15	2.1	1.87	1.75	8.88
			35.5	23.7	21.05	19.74	
			4.5	10.2	30.77	53.57	
		Total	600	176	52	28	856
			70.1	20.6	6.07	3.27	100

Agreement between patient (24 month) and physician (36 month) is 63.0%

Weighted Kappa = 0.3664 (0.31, 0.42)

### Risk model of severe PTS

**Fig. 3:**
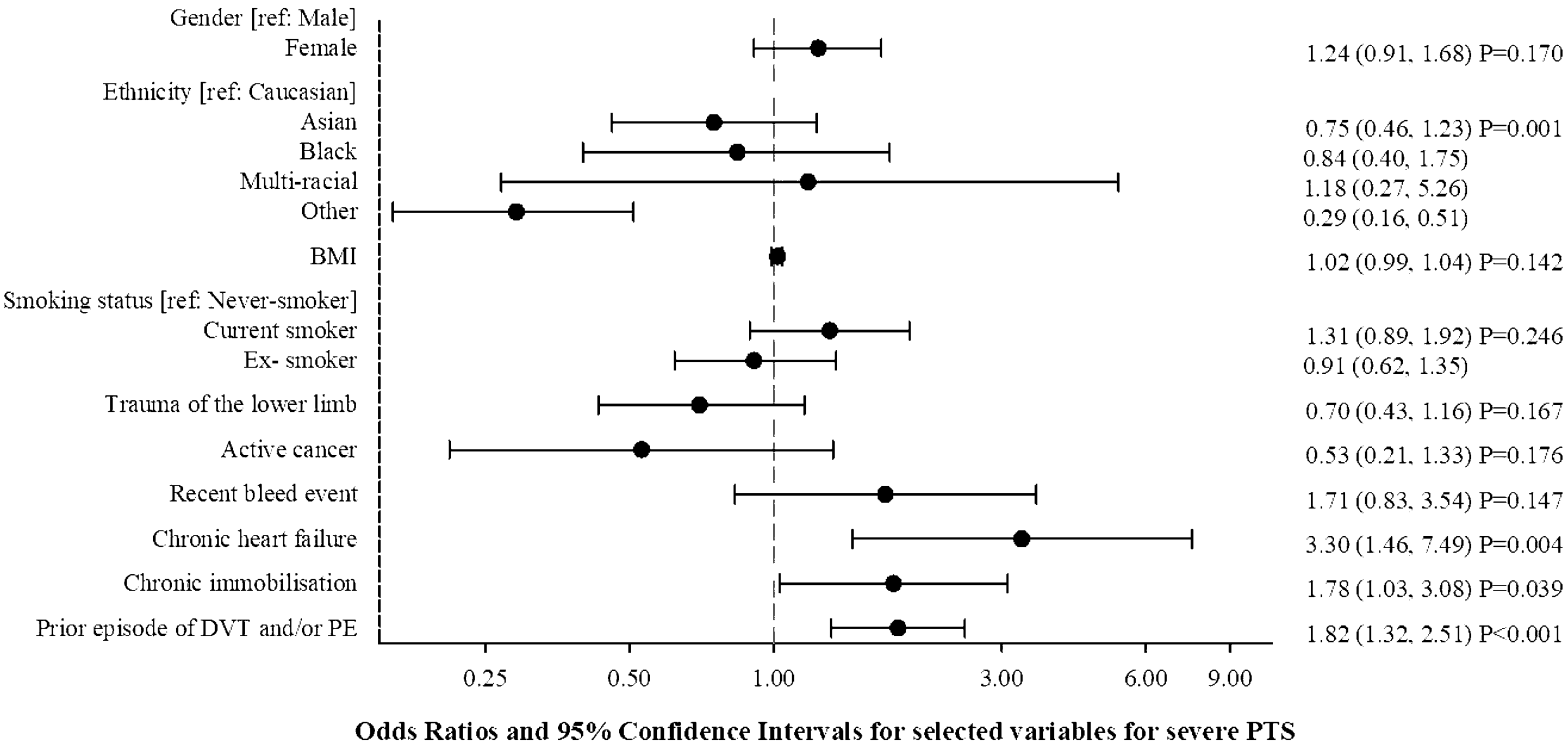
Selected variables for severe PTS multivariable model with corresponding odd ratios (OR) 95% CI, and p values

**Fig. 4:**
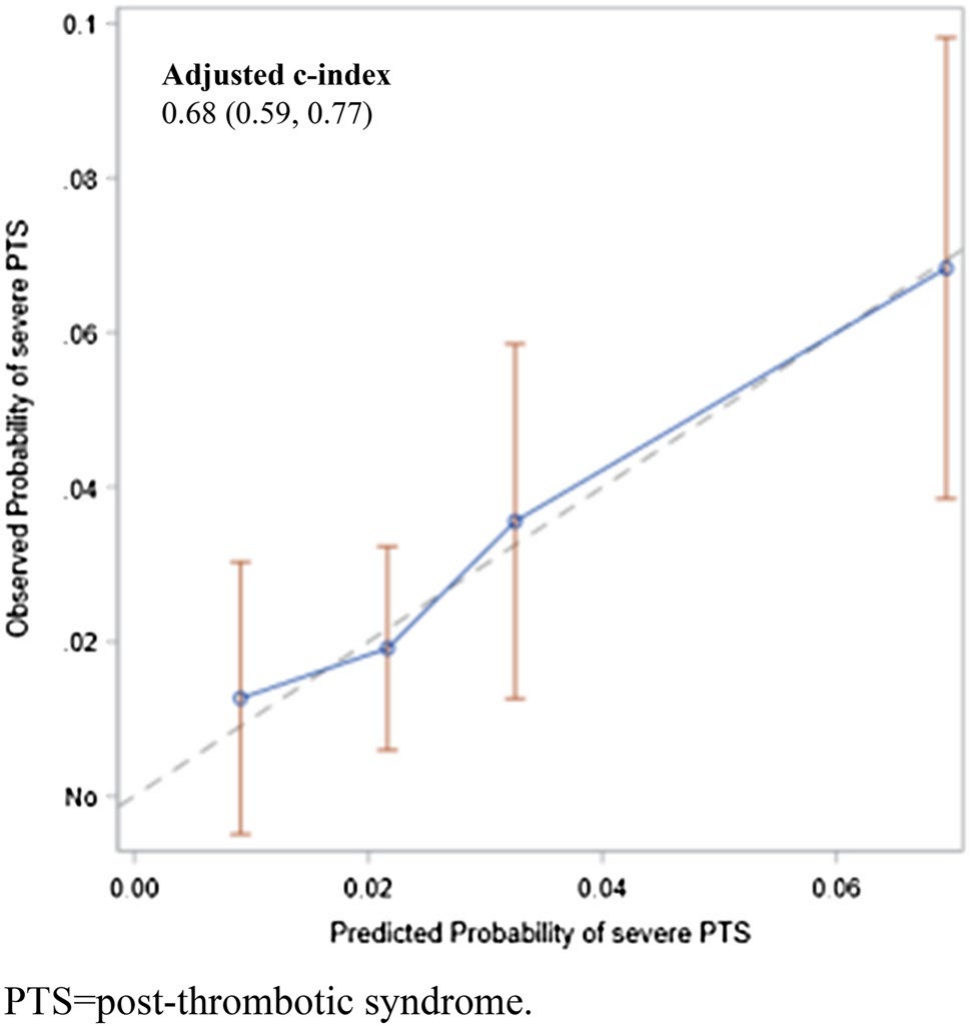
Calibration curve for predicted versus observed severe PTS. *PTS* post-thrombotic syndrome. The parametric p value is calculated by ANOVA for numerical covariates and chi-square test for categorical covariates

Figure 3 (see Supplementary Table 2 for full results) shows the results of the multivariable analysis that was conducted to identify parameters associated with the risk of severe PTS. As shown in the figure, chronic heart failure, chronic immobilization, and prior episode of DVT were found to be independent predictors of severe PTS. The multivariable model provided good discrimination for severe PTS over 36-months, yielding an optimistic adjusted c-index of 0.68 (95% CI 0.59 to 0.77) (Fig. 4).

## Discussion

The management of PTS can be both time-consuming and frustrating for clinicians. Once established, especially when complicated with leg ulceration, it is a significant cause of disability and economic burden for patients and healthcare systems alike [10, 11].

Proximal DVT is known to be associated with a higher frequency and more severe PTS than distal DVT [2]. Typically studies in PTS have focused on these patients and for the results to this analysis to be comparable to similar studies only patients with proximal DVT were included. The key findings of this analysis which includes both proximal and distal DVT patients have previously been presented in ISTH 2021 [12].

Based on the experience gained in contemporary years in the framework of the international GARFIELD-VTE registry, the rate of both overall and severe PTS, as assessed by physicians 3 years after the thrombotic episode, seems remarkably lower than that reported in past prospective cohorts and randomized clinical trials [1, 2]. Indeed, overall PTS was found in approximately one quarter of patients (as compared to 40 to 50%), and that of the most severe manifestations in under 4% (as compared to 8 to 10%). Of course, the heterogeneity of data collection as performed by untrained physicians in the real world can account at least partially for this discrepancy. When examining the change in patient self-reported Villalta from 6 to 24 months a 63% agreeance between time frames was observed. This is a similar level of agreeance we see between patients with a 24-month and 36-month physician score. It is important to note that differences in individual categories of PTS (none, moderate, mild, severe) will have more variability than observing binary presence or absence of PTS. However, the fair correspondence between the physician’s assessment and the patient’s self-evaluation demonstrated by the weighted Kappa of 0.3664 (0.31, 0.42) makes these findings reliable. It should be considered that referral of patients with suspected DVT to specialists and the diagnostic process has seen major improvements over time, as documented by a shortening of patient-doctor delay and the replacement of high-threshold, invasive contrast venography by non-invasive and easily accessible ultrasonography, besides the improved initial and long-term treatment of DVT, including the replacement of VKA with the novel DOACs and the refinement of long-term anticoagulation strategies. Hence, it is likely that in recent years the thrombotic burden of patients with DVT has considerably reduced in comparison to older studies.

Patients with PTS were on average older and had a higher prevalence of prior episodes of VTE. They also had a lower prevalence of transient risk factors of DVT and were more likely to have persistent risk factors of DVT, such as chronic heart failure and chronic immobilization. It is also worth noting that special patient populations such as those that have renal insufficiency or a history of cancer did not appear more likely to have PTS. It is possible that this may be due to the low patient numbers and poorer survival of these patients, however further investigation in these patient populations may be warranted. These findings are consistent with those coming from most contemporary studies [6, 7]. Some of the risk factors detailed in this analysis have not been commonly mentioned in previous studies but recorded in the GARFIELD-VTE registry after consultation from clinical experts in the field. Clinical risk factors that have been commonly associated with increased risk of PTS include obesity, chronic heart failure and a history of VTE [13–15], however in another large VTE registry (COMMAND-VTE) chronic kidney disease, leg swelling, and active cancer were independent risk factors for PTS [16].

For detecting and grading the severity of PTS we adopted the Villalta score, which among available tools is by far the most widely used and recommended by scientific societies [5]. Diagnosis of PTS by the Villalta score requires a clinical visit to perform a physical examination of the affected limb, which represents a challenge and limits the possibility of its use when conducting large studies as well as in several circumstances, such as the current pandemic. Furthermore, this may be considered an interventional procedure as it requires an examination of the patient that is not normally performed in routine patient care and is therefore not suitable for prospective non-interventional registry studies. While a tool for patient reporting of the Villalta score would reduce health resource utilization and the burden imposed on patients participating in clinical trials, the questionnaires so far developed and validated for patient self-assessment require a visually assisted form [5, 17]. In our project, a patient’s self-evaluation was obtained after 2 years with the help of a questionnaire enquiring the presence and severity of subjective symptoms and objective findings (all but skin ulcer). Although all items were completed by the patient without any visual support, the self-assessment of overall PTS at 24-months was found to be accurate (accuracy, 0.74; 95% CI 0.71 to 0.76) with respect to the physician’s 36-month evaluation, with a good sensitivity and specificity (66 and 77%, respectively). Hence, our findings have the potential to provide clinicians with a new useful tool for detecting PTS and quantifying its severity without the aid from health-care personnel.

Although in recent years a few scores have been identified and validated that can help predict the development of PTS [18–21], their value is uncertain, as is their potential implications for clinical practice [7]. Based on the results of the multivariable analysis we have conducted, chronic heart failure and chronic immobilization were found to be statistically significant independent predictors of severe PTS, while there was a weak association between the use of compression stockings and reduced likelihood of PTS. This simple and practical multivariable model provided good discrimination for severe PTS over 36-months, yielding an optimistic adjusted c-index of 0.61 (95% CI 0.58 to 0.64). Accordingly, it has the potential to help predict the development of severe PTS even in the hands of less experienced physicians. However, its implementation in clinical practice requires external validation from additional cohorts of patients.

Our investigation presents several limitations that deserve careful attention. The clinical examination regarding PTS was conducted by physicians who represent the standard of care in the respective countries, but who, in contrast to randomised clinical trials, were not specifically trained for the PTS examination. In order to prevent misinterpretation of clinical signs, the reporting of skin ulcer was not included in the questionnaire addressing the patient’s self-assessment of PTS. Furthermore, additional well-known risk factors of PTS, such as the ilio-femoral location of thrombosis and the presence of varicose veins or other manifestations of chronic venous insufficiency were not captured by the registry, nor was the development of ultrasound detectable manifestations of vascular damage (residual vein thrombosis or popliteal valve reflux). Since heart failure is associated with an increased risk of PTS another limitation that should be noted is that there is a risk that venous insufficiency and heart failure symptoms could be misdiagnosed as PTS.

For logistic reasons, it was not possible to obtain a patient’s self-assessment of PTS after 3 years, i.e. at the same time of the physician’s assessment. However, it is well known that most PTS complications develop within the first 2 years of the thrombotic episode [6]. Finally, the prescription of elastic stockings was left to discretion of the investigator and no information is available on the type and duration of these devices.

In conclusion, our findings suggest that the incidence and severity of PTS in the real world is lower than that reported in contemporary studies addressing the development of this complication. The patient self-assessment we described for the first time using the Villalta score at 24 months can be used to simply estimate the risk of PTS over 36 months. A simple multivariate risk model, based on easily accessible information at patient’s presentation provided good discriminatory power for predicting severe PTS risk over 36 months after DVT diagnosis.

### Supplementary Information

Below is the link to the electronic supplementary material.

Supplementary file1 (DOCX 30 KB)

Supplementary file2 (DOCX 25 KB)

## Data Availability

Aggregated data can be shared upon reasonable request and analysis plan to Saverio Virdone (Svirdone@tri-london.ac.uk).
