# Supplementary material for: Incidence and predictors of post-thrombotic syndrome in patients with proximal DVT in a real-world setting: findings from the GARFIELD-VTE registry
Source: J Thromb Thrombolysis. 2023 Nov 6;57(2):312–21. doi: 10.1007/s11239-023-02895-7 (PMC10869374; doi:10.1007/s11239-023-02895-7)
Supplement: Supplementary file 1 — Supplementary file1 (DOCX 30 KB) [file 11239_2023_2895_MOESM1_ESM.docx]

**Supplementary material**

**Supplementary table 1: Original Villalta score^1^**

| Symptoms and clinical signs | None | Mild | Moderate | Severe |
| --- | --- | --- | --- | --- |
| Symptoms |  |  |  |  |
| Pain | 0 points | 1 point | 2 points | 3 points |
| Cramps | 0 points | 1 point | 2 points | 3 points |
| Heaviness | 0 points | 1 point | 2 points | 3 points |
| Paresthesia | 0 points | 1 point | 2 points | 3 points |
| Pruritus | 0 points | 1 point | 2 points | 3 points |
| Clinical signs | 0 points | 1 point | 2 points | 3 points |
| Clinical signs |  |  |  |  |
| Pretibial edema | 0 points | 1 point | 2 points | 3 points |
| Skin induration | 0 points | 1 point | 2 points | 3 points |
| Hyperpigmentation | 0 points | 1 point | 2 points | 3 points |
| Redness | 0 points | 1 point | 2 points | 3 points |
| Venous ectasia | 0 points | 1 point | 2 points | 3 points |
| Pain on calf compression | 0 points | 1 point | 2 points | 3 points |
| Venous ulcer | Absent | Present |  |  |

^1^Kahn SR et al, J Thromb Haemost 2009; 7(5):879-83​.

## Supplementary table 2: Selected variables for severe PTS multivariable model with corresponding odd ratios (OR), 95% CI and Wald Chi-square test.

| **Variable** | **OR** | **95% CI** | **DF** | **Chisq** | **P-Value** |
| --- | --- | --- | --- | --- | --- |
| Acute Medical Illness | 2.46 | (0.79, 7.94) | 1 | 2.25 | 0.134 |
| Trauma of the Lower Limb | 0.26 | (0.03, 1.95) | 1 | 1.73 | 0.189 |
| Hospitalisation | 0.38 | (0.08, 1.85) | 1 | 1.44 | 0.230 |
| Chronic Heart Failure | 5.28 | (1.69, 16.54) | 1 | 8.17 | **0.004** |
| Chronic immobilisation | 3.25 | (1.19, 9.32) | 1 | 4.83 | **0.028** |
| Prior episode of DVT and/or PE | 2.04 | (0.99, 4.17) | 1 | 3.76 | **0.053** |
| Compression therapy | 0.66 | (0.39, 1.27) | 1 | 1.57 | 0.211 |

CI, confidence interval; DF, degrees of freedom; Chisq. Chi square.

**Supplementary table 3: Baseline characteristics of patients deceased before recording of physician’s score.**

| **Baseline characteristic** | **Level** | **Physician follow-up=1107** | **Deceased N=1175** | **P-value** |
| --- | --- | --- | --- | --- |
| Age (years) | Overall | 1107 | 1175 | **<0.001** |
|  | Male | 56.1 (16.5) | 66.8 (14.4) |  |
|  | Female | 57.6 (43.6;68.6) | 69.0 (58.2;77.1) |  |
| BMI (kg/m²) | Overall | 1055 | 1075 | **<0.001** |
|  | Male | 29.0 (6.2) | 26.6 (6.9) |  |
|  | Female | 27.9 (24.8;32.0) | 25.7 (22.2;29.4) |  |
| Sex | Male | 597 (53.9) | 573 (48.8) | **0.014** |
|  | Female | 510 (46.1) | 602 (51.2) |  |
| Ethnicity/race | Asian | 89 (8.7) | 356 (31.6) | **<0.001** |
|  | Black | 36 (3.5) | 82 (7.3) |  |
|  | Caucasian | 777 (75.8) | 637 (56.5) |  |
|  | Multi-racial | 6 (0.6) | 5 (0.4) |  |
|  | Other | 117 (11.4) | 47 (4.2) |  |
| Smoking status | Never smoker | 697 (63.9) | 668 (58.4) | **<0.001** |
|  | Ex-smoker | 200 (18.3) | 332 (29.0) |  |
|  | Current smoker | 194 (17.8) | 144 (12.6) |  |
| Treatments | Parenteral therapy only | 135 (12.2) | 464 (40.0) | **<0.001** |
|  | Parenteral therapy + VKA | 357 (32.3) | 260 (22.4) |  |
|  | VKA only | 51 (4.6) | 71 (6.1) |  |
|  | DOAC only | 385 (34.8) | 195 (16.8) |  |
|  | Parenteral therapy + DOAC | 163 (14.7) | 110 (9.5) |  |
|  | Other AC | 5 (0.5) | 24 (2.1) |  |
|  | No AC treatment | 10 (0.9) | 33 (2.8) |  |
|  | Dead/Withdrawn/Lost to follow up | 0 (0.0) | 3 (0.3) |  |
| Provoking risk factors | Compression therapy | 717 (64.8) | 303 (25.8) | **<0.001** |
|  | Acute Medical Illness | 61 (5.5) | 126 (10.7) |  |
|  | Hospitalisation | 92 (8.3) | 262 (22.3) | **<0.001** |
|  | Long-Haul Travelling | 50 (4.5) | 23 (2.0) |  |
|  | Trauma of the Lower Limb | 113 (10.2) | 29 (2.5) | **<0.001** |
|  | Surgery | 95 (8.6) | 136 (11.6) |  |
|  | Active Cancer | 35 (3.2) | 458 (39.0) | **<0.001** |
| Persistent/predisposing risk factors | Chronic Heart Failure | 26 (2.3) | 96 (8.2) | **<0.001** |
|  | Chronic immobilisation | 64 (5.8) | 122 (10.4) |  |
|  | Family history of VTE (first degree relatives) | 62 (5.6) | 24 (2.0) |  |
|  | History of Cancer | 85 (7.7) | 401 (34.1) | **<0.001** |
|  | Known thrombophilia | 41 (3.7) | 17 (1.4) |  |
|  | Prior episode of DVT and/or PE | 218 (19.7) | 113 (9.6) |  |
|  | Renal insufficiency | 28 (2.5) | 112 (9.5) | **<.001** |

^*^ The parametric p-value is calculated by ANOVA for numerical covariates and chi-square test for categorical covariates.
