# Supplementary material for: Incidence and predictors of post-thrombotic syndrome in patients with proximal DVT in a real-world setting: findings from the GARFIELD-VTE registry
Source: J Thromb Thrombolysis. 2023 Nov 6;57(2):312–21. doi: 10.1007/s11239-023-02895-7 (PMC10869374; doi:10.1007/s11239-023-02895-7)
Supplement: Supplementary file 2 — Supplementary file2 (DOCX 25 KB) [file 11239_2023_2895_MOESM2_ESM.docx]

**Appendix:**

**A full list of GARFIELD-VTE Principal Investigators**

Abdullah,Nik; Abiko,Akihiko; Abril,Juan; Acevedo,David; Adademir,Taylan; Adler,David;

Ageno,Walter; Agnelli,Giancarlo; Ahmed,Mostafa; Aksoy,Ahmet; Aktogu,Serir; Ali,Gholam; Alikhan,Raz; Allen,Gregory; Angchaisuksiri,Pantep; Antoinette,Sevestre; Arouni,Amy; Azeddine,Addala; Azim,Tarek; Backer,Wilfried; Balthazar,Yohan; Bang,Soo; Banyai,Martin; Barbarash,Olga; Barrionuevo,Marcelo; Bary,Mostafa; Battaloglu,Bektas; Bax,W; Béatrice,Terriat; Behrens,Steffen; Belenky,Dmitry; Benitez,Juan; Berli,Mario; Bernadette,Peuch; Berni,Andrea; Betsbrugge,Michiel; Beyers,Adriaan; Bezuidenhout,Abraham; Bidi,Claude; Bilderling,Peter; Binet,Laure; Biss,Tina; Blasco,Luis; Blessing,Erwin; Blombery,Peter; Bono,Julio; Boomars,Karin; Boondumrongsagoon,Juree; Borges,Lohana; Bosch,Manuel; Botha,Louis; Bounameaux,Henri; Boussy,Tim; Bowers,Margaret; Boyarkin,Mikhail; Brauer,Cornelia; Burbury,Kate; Burianova,Hana; Burov,Yuriy; Cader,Cas; Canevascini,Reto; Capiau,Luc; Cappelli,Roberto; Carine,Boulon; Carrier,Marc; Carrim,Abu; Carroll,Patrick; Casabella,Tomas; Cate,Hugo; Cattaneo,Marco; Cech,Vladimir; Cervera,Luis; Cha,Seung; Chacko,Joseph; Chang,Kuan; Chansung,Kanchana; Chao,Ting; Chauhan,Anoop; Chayangsu,Sunee; Chetanachan,Mariam; Chew,Lee; Chiang,Chern; Chiu,Kuan; Choi,Won; Christian,Ponchaux; Christophe,Brousse; Christophe,Seinturier; Chunilal,Sanjeev; Clark,Amanda; Colak,Abdurrahim; Correa,João; Cosmi,Benilde; Cosmi,Franco; Coufal,Zdenek; Creagh,Desmond; Cristina,Leone; Cuneo,Carlos; Dalmau,Garcia; Damien,Garrigues; D'Angelo,Armando; Darius,Harald; Datta,Sudip; Dees,Adriaan; Dessoki,Mohamed; Diaz,Carlos; Diaz,Enrique; Dogan,Emre; Dominique,Brisot; Dominique,Elkouri; Dominique,Stephan; Donders,Servaas; Dorokhov,Dmitry; Duchateau,Johan; Duda,Norberto; Eddie,Grace; Elali,Hallah; ElDin,Hesham; Elisa,Chevrier; Emmanuel,Messas; Erdelyi,Barbara; Erdkamp,Frans; Esheiba,Ehab; Esperón,Guillermo; Essameldin,Sherif; Everington,Tamara; Faghih,Markus; Falanga,Anna; Fedele,Jose; Ferkl,Richard; Fernandez,Alberto; Fernandez,Manuel; Ferrini,Piera; Ferroni,Fabian; Filho,Jose; Fixley,Mark; Fletcher,John; Flores,Oscar; Francis,Couturaud; Francois,Bergmann; Franow,Hendrik; Gad,Amr; Gaffar,Mohamed; Gaffney,Mary; Gal,Gregoire; Galvar,Javier; Galvez,Angel; Gamba,Marco; Gan,Gin; Gerdes,Victor; Gerofke,Hagen; Gibbs,Harry; Gogia,Harinder; Gordeev,Ivan; Goto,Shinya; Griffin,Sam; Gris,Christina; Grochenig,Ernst; Gujral,Jaspal; Gur,Ozcan; Gurbuz,Orcun; Gustin,Michel; Guzman,Luis; Ha,Chung; Haddad,Ghassan; Hagemann,Dirk; Hainaut,Philippe; Hameed,Muhammad; Hart,Terence; Hasanoglu,Hatice; Hashas,Erman; Haverkamp,Wilhelm; Helene,Desmurs; Henry,Fitjerald; Herdy,Artur; Herreweghe,Rika; Hirano,Masao; Ho,Prahlad; Ho,Wai; Hollanders,Geert; Homza,Miroslav; Horacek,Thomas; Hsia,Chien; Huang,Chien; Huang,Chi; Huang,Chun; Humphrey,Julian; Hunt,Beverley; Husin,Azlan; Hwang,Hun; Iamsai,Piriyaporn; Ibarra,Manuel; Imberti,Davide; Isabelle,Mahe; Isbir,Selim; Jacobson,Barry; Jansky,Petr; Jiang,Weihong; Jimenez,David; Jing,Zhicheng; Jing,Zhicheng; Joh,Jin; Kamalov,Gadel; Kanda,Junji; Kanemoto,Masashi; Kanitsap,Nonglak; Kanko,Muhip; Karaarslan,Kemal; Kassis,Jeannine; Kato,Atsushi; Kazakov,Andrey; Keeling,David; Keim,Reinhold; Kelly,Allan; Khan,Mohamed; Kho,Bonnie; Khotuntsov,Alexey; Kim,Ho; Kim,Igor; Kim,JangYong; Kim,Jin; Kim,Moo; Kim,Yang; Kiris,Ilker; Klamroth,Robert; Kleiban,Andres; Klein,Garry; Kondo,Katsuhiro; Koretzky,Martin; Korte,Wolfgang; Koto,Modise; Koura,Firas; Kovacs,Michael; Krasavin,Vladimir; Krichell,Alan; Kroeger,Knut; Kroening,Ralf; Krupicka,Jiri; Kubat,Emre; Kucera,Dusan; Kuki,Shintaro; Kuo,Jen; Kvasnicka,Jan; Kwok,Chi; Kwon,JiHyun; Lai,Wen; Lang,Pavel; Lara,Jose; lastfirst; Lastuvka,Jiri; Lawall,Holger; Leahy,Michael; Lee,Jae; Lee,Moon; Leon,Raul; Léopold,Siwe; Levy,Michael; Libov,Igor; Lin,Wei; Lockman,Ann; Lodigiani,Corrado; Looi,Irene; López,Luciano; Loualidi,Ab; Lunn,Charles; Luo,Canhua; Luvhengo,Thifhelimbilu; Maasdorp,Shaun; MacCallum,Peter; Machowski,Andrew; Majumder,Mujibur; Makruasi,Nisa; Malek,Wagih; Manuel,Kubina; Marchena,Pablo; Marino,Javier; Martinez,Rafael; Matsuoka,Shunzo; Mazzone,Antonino; McRae,Simon; Mellor,Stuart; Mendes,Robert; Merli,Geno; Mestre,Antoni; Michèle,Escande; Middeldorp,Saskia; Miranda,Raimundo; Mohamed,Ahmed; Mohamed,Monniaty; Moia,Marco; Møller,Dorthe; Motte,Serge; Moustafa,Moustafa; Mumoli,Nicola; Mun,Yeung; Munch,Michael; Muntaner,Juan; Mustafa,Bisher; Mutirangura,Pramook; Myriam,Martin; Na,Sang; Nagib,Mohamed; Nakamura,Hiroaki; Nakamura,Mashio; Nakazawa,Satoshi; Nam,Seung; Natha,Bhavesh; Nicolas,Falvo; Nielsen,Jørn; Norasetthada,Lalita; Nordin,Nordiana; Numbenjapon,Tontanai; Nyvad,Ole; Ohler,Hans; Ohnuma,Yasushi; Olsen,Michael; Onodera,Tomoya; Opitz,Christian; Oropallo,Alisha; Otero,Remedios; Oto,Oztekin; Paez,Jorge; Panchenko,Elizaveta; Paredes,Félix; Park,Jin; Park,Yong; Paruk,Nishen; Patanasing,Siriwimon; Paul,Guillot; Pauw,Michel; Peromingo,Jose; Petrov,Dmitry; Pharr,Walter; Plassmann,Georg; Platt,George; Podpera,Ivo; Poirier,Germain; Poli,Daniela; Porreca,Ettore; Prisco,Domenico; Prosecky,Robert; Pumprla,Jiri; Raedt,Herbert; Ratsela,Rapule; Raymundo,Selma; Reyes,Raquel; Reynolds,Tim; Ria,Luigi; Rojnuckarin,Ponlapat; Roux,Dirk; Salem,Ayman; Santoro,Rita; Saraiva,Jose; Sathar,Jameela; Savas,Ismail; Schellong,Sebastian; Schiavi,Lilia; Schmidt,Andor; Schmidt,Renate; Schroe,Herman; Schul,Marlin; Schwencke,Carsten; Scott,David; Shah,Gaurand; Shibata,Yoshisato; Shih,Jhih; Shim,Hyeok; Sholkamy,Sherif; Shyu,Kou; Singh,Rupesh; Singh,Suaran; Skowasch,Dirk; Slocombe,Alison; Smith,Clifford; Sokurenko,German; Soliman,Mosaad; Solymoss,Susan; Song,Ik; Sonkin,Igor; Souto,Joan; Spacek,Rudolf; Staroverov,Ilya; Staub,Daniel; Striekwold,Harry; Stuecker,Markus; Subbotin,Yuriy; Suchkov,Igor; Sun,Shenghua; Surinach,Jose; Suwanban,Tawatchai; Svatopluk,Koscál; Svobodova,Jaromira; Tahar,Mersel; Takeuchi,Kensuke; Tanabe,Yasuhiro; Tenorio,Isabel; Testa,Sophie; Theodoro,Daniel; Tian,Hongyan; Tick,Lidwine; Timmermans,Luc; Ting,Seng; Tiraferri,Eros; Toh,Cheng; Toh,See; Tolstikhin,Vladimir; Toro,Jorge; Toro,Jorge; Tosetto,Alberto; Toufek,Berremeli; Trimarco,Bruno; Tse,Eric; Tseng,Wei; Turker,Hatice; Ueng,Kwo; Usandizaga,Esther; Vandenbosch,Kristel; Vanwelden,Jan; Verhamme,Peter; Vesely,Jiri; Vesti,Beatrice; Viboonjuntra,Pongtep; Vilamajo,Oscar; Vleeschauwer,Philippe; Wang,Haofu; Wang,Shenming; Ward,Chris; Watanabe,Akinori; Watt,Simon; Welker,James; Wells,Rachel; Wern,Kwan; Westendorf,Jan; White,Richard; Wilson,Benedicte; Wong,Lily; Wong,Raymond; Wongkhantee,Somchai; Wu,Chau; Wu,Chih; Wu,Cynthia; Yang,Jinghua; Yang,Zhenwen; Yang.,Zhongqi; Yavuz,Celal; Yeo,Erik; Yhim,Ho; Yiu,Kai; Yoshida,Shuichi; Yoshida,Winston; Zaidman,Cesar; Zateyshchikov,Dmitry; Zeller,Thomas; Zemek,Stanislav; Zhang,Lei; Zhang,Weihua; Zhu,Hong; Zidan,Hesham; Zidel,Brian; Zrazhevskiy,Konstantin; Zubareva,Nadezhda;
